# Structural insights into catalytical capability for CPT11 hydrolysis and substrate specificity of a novel marine microbial carboxylesterase, E93

**DOI:** 10.3389/fmicb.2022.1081094

**Published:** 2023-01-11

**Authors:** Yang Li, Zhen Rong, Zhengyang Li, Henglin Cui, Jixi Li, Xue-Wei Xu

**Affiliations:** ^1^School of Oceanography, Zhejiang University, Zhoushan, China; ^2^Key Laboratory of Marine Ecosystem Dynamics, Ministry of Natural Resources and Second Institute of Oceanography, Ministry of Natural Resources, Hangzhou, China; ^3^State Key Laboratory of Genetic Engineering, School of Life Sciences, Shanghai Engineering Research Center of Industrial Microorganisms, Fudan University, Shanghai, China; ^4^School of Food and Biological Engineering, Jiangsu University, Zhenjiang, China

**Keywords:** marine bacterial carboxylesterase, crystal structure, enzyme catalysis, prodrug, p-nitrophenyl, substrate specificity

## Abstract

**Introduction:**

CPT11 (Irinotecan; 7-ethyl-10-[4-(1-piperidino)-1-piperidino] carbonyloxycamptothecin) is an important camptothecin-based broad-spectrum anticancer prodrug. The activation of its warhead, SN38 (7-ethyl-10-hydroxycamptothecin), requires hydrolysis by carboxylesterases. NPC (7-ethyl-10-[4-(1-piperidino)-1-amino] carbonyloxycamptothecin) is a metabolic derivative of CPT11 and is difficult to be hydrolyzed by human carboxylesterase. Microbial carboxylesterase with capability on both CPT11 and NPC hydrolysis is rarely reported. A marine microbial carboxylesterase, E93, was identified to hydrolyze both substrates in this study. This enzyme was an appropriate subject for uncovering the catalytic mechanism of carboxylesterases to CPT11 and NPC hydrolysis.

**Methods:**

X-ray diffraction method was applied to obtain high-resolution structure of E93. Molecular docking was adopted to analyze the interaction of E93 with *p*-NP (*p*-nitrophenyl), CPT11, and NPC substrates. Mutagenesis and enzymatic assay were adopted to verify the binding pattern of substrates.

**Results:**

Three core regions (Region A, B, and C) of the catalytic pocket were identified and their functions on substrates specificity were validated *via* mutagenesis assays. The Region A was involved in the binding with the alcohol group of all tested substrates. The size and hydrophobicity of the region determined the binding affinity. The Region B accommodated the acyl group of *p*-NP and CPT11 substrates. The polarity of this region determined the catalytic preference to both substrates. The Region C specifically accommodated the acyl group of NPC. The interaction from the acidic residue, E428, contributed to the binding of E93 with NPC.

**Discussion:**

The study analyzed both unique and conserved structures of the pocket in E93, for the first time demonstrating the discrepancy of substrate-enzyme interaction between CPT11 and NPC. It also expanded the knowledge about the substrate specificity and potential application of microbial Family VII carboxylesterases.

## 1. Introduction

Carboxylesterase (EC 3.1.1.1), which catalyzes the formation of ester bonds in the organic phase and the cleavage of ester bonds in the aqueous phase, is widely distributed in microorganisms, plants, and animals (Wierdl et al., 2004; Satoh and Hosokawa, 2006; Zhu et al., 2013; Wang et al., 2020). Carboxylesterase in human is the major enzyme for ester prodrug activation (Redinbo and Potter, 2005; Ross et al., 2006; Ross and Crow, 2007; Hosokawa, 2008; Zhu et al., 2013). Ester prodrug is a category of synthetic compounds where the drug warhead and the chemically modified group is connected *via* ester bonds. The drug warhead can be released by the carboxylesterase-mediated ester bond hydrolysis, and pharmacological effects of the ester prodrug can then be activated (Stubdal et al., 2003; Xie et al., 2003; Lavis, 2008). Ester prodrug is the most widely used prodrug category in anti-cancer medication (Rautio et al., 2008).

**Figure 1:**
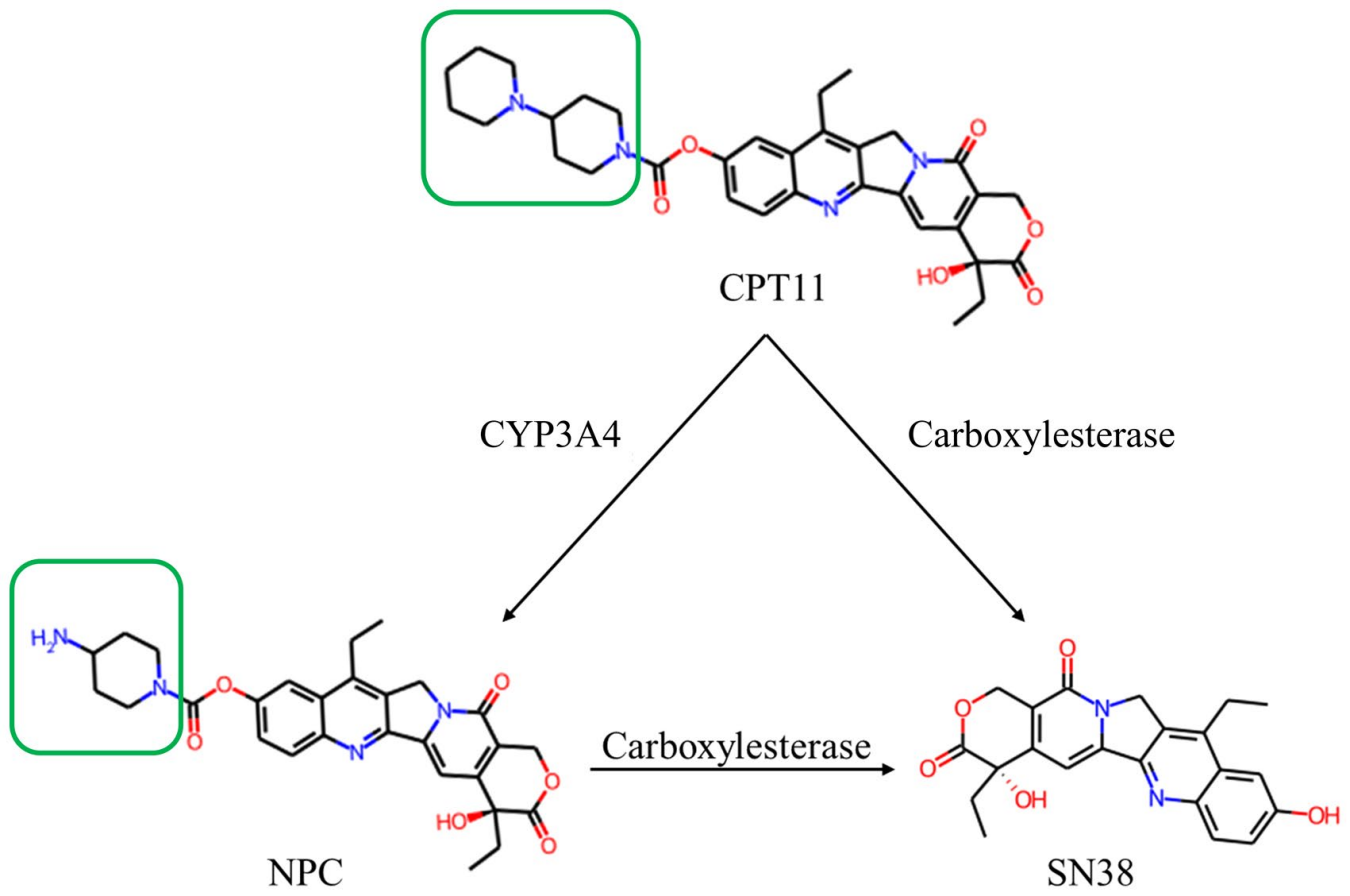
Structure and relationship demonstration of CPT11, NPC and SN38. CPT11 is oxidized by cytochrome P450 3A4 (CYP3A4) isoenzyme to produce 7-ethyl-10-[4-(1-piperidino)-1-amino]-carbonyloxycamptothecin (NPC). CPT11 are metabolized by carboxylesterases (CES) to produce active metabolite, 7-ethyl-10-hydroxycamptothecin (SN38). The only structural difference between CPT11 and NPC is marked with the green box.

CPT11 (7-ethyl-10-[4-(1-piperidino)-1-piperidine] carbonyloxy-camptothecin, irinotecan) is a classical carbamate anticancer prodrug with molecular modifications *via* an ester bond. It shows significant antitumor activity in clinical trials for a broad spectrum of cancers (Reita et al., 2019), including non-small cell lung (Zhang et al., 2015), pancreatic (Tossey et al., 2019), and gastric cancers (Fukuchi et al., 2020). Therefore, CPT11 remain the main anticancer drugs for clinical use. The antitumor function of CPT11 is achieved when it is converted to the active compound SN38 (7-ethyl-10-hydroxyamptothecin) through endogenous carboxylesterase-catalyzed hydrolysis of the ester bond (Pommier, 2006; Gentry et al., 2011; Figure 1). One major human liver carboxylesterase (EC 3.1.1.1), hCES2, is involved in ester bond hydrolysis and conversion of CPT11 into SN38 (Santos et al., 2000; Sanghani et al., 2004). In medication practice, the pharmacological effects of CPT11 are partly determined by the individual differences of hCE2 expression. The anti-cancer function of CPT11 might not be fully achieved to the patients with comparatively low hCE2 expression level (Jewell et al., 2007). Enzyme-Prodrug Therapy (EPT) is one of strategies to avoid the individual difference during the medication. The EPT adopts and introduces heterogenous carboxylesterase into the tumor cells at lesion location. The identification and analysis of heterogenous carboxylesterase that can active prodrug would be meaningful to the development of therapy (Schellmann et al., 2010; Lehouritis et al., 2013). Microbial enzymes with capability of prodrug activation are particularly promising exogenous enzymes (Lehouritis et al., 2013).

During metabolism in humans, part of CPT11 could be also hydrolyzed by cytochrome P450 3A4 (CYP3A4) to 7-ethyl-10-[4-amino-1-piperidinyl] carbonyl oxycamptothecin (NPC), the major by-product of CPT11 (Santos et al., 2000; Figure 1). NPC is barely converted to SN38 by human carboxylesterases, leading to the loss of pharmaceutical efficacy (Santos et al., 2000; Sanghani et al., 2004). There was no relevant report on microbial carboxylesterase activating NPC. The structural mechanism of carboxylesterase-mediated CPT11 and NPC hydrolysis is not clear.

In this study, the microbial family VII carboxylesterase E93 was identified from the marine bacterium *Altererythrobacter indicus* DSM 18604. Enzymatic assay identified its catalytic activity to the hydrolysis of both CPT11 and NPC substrates. The structure of E93 was obtained *via* crystallography and X-ray diffraction. The catalytic pocket was divided by catalytic triad into three major regions (Region A-C). By comparing the catalytic pocket with that from the same carboxylesterase family, the structure of each region was discussed. *Via* enzymatic assay with different substrates (CPT11, NPC, and the general carboxylesterase substrate *p*-NP ester), the large-sized residues in region A, polar residues in the region B, and an acidic residue in the region C, were identified to be involved in the substrate interaction and selection. The analysis showed that CPT11 and NPC had different binding feature to carboxylesterase E93. This study contributes to understanding the structural mechanism of substrate preference of family VII carboxylesterases and the hydrolysis of camptothecin-like drug substrates. It may facilitate the use of carboxylesterases in medical treatment.

## 2. Results

### 2.1. Amino acids sequence of E93

E93 was identified from the marine bacterium *A. indicus* DSM 18604 (Seo and Lee, 2010). The amino acids sequence had high identity with putative microbial carboxylesterases, including WP_237437685.1 and WP_191229314.1 (92 and 76% in identity). However, E93 had less than 45% similarity to carboxylesterases from territorial microorganisms. The sequential alignment in the ESTHER database categorized E93 into the Carb_B family esterase, where all reported Family VII carboxylesterases belong to (Lenfant et al., 2013).

Phylogenetic analysis showed close evolutionary relationships between E93 and family VII esterases (Arpigny and Jaeger, 1999), including carboxylesterase from *B. subtilis* (P37967), phenmedipham hydrolase from *Arthrobacter oxydans* (Q01470), and putative carboxylesterase from *Streptomyces coelicolor* A3 (CAA22794; Supplementary Figure S1). It indicated that E93 was a novel microbial family VII esterase member.

### 2.2. Biochemical characterization of E93

**Figure 2:**
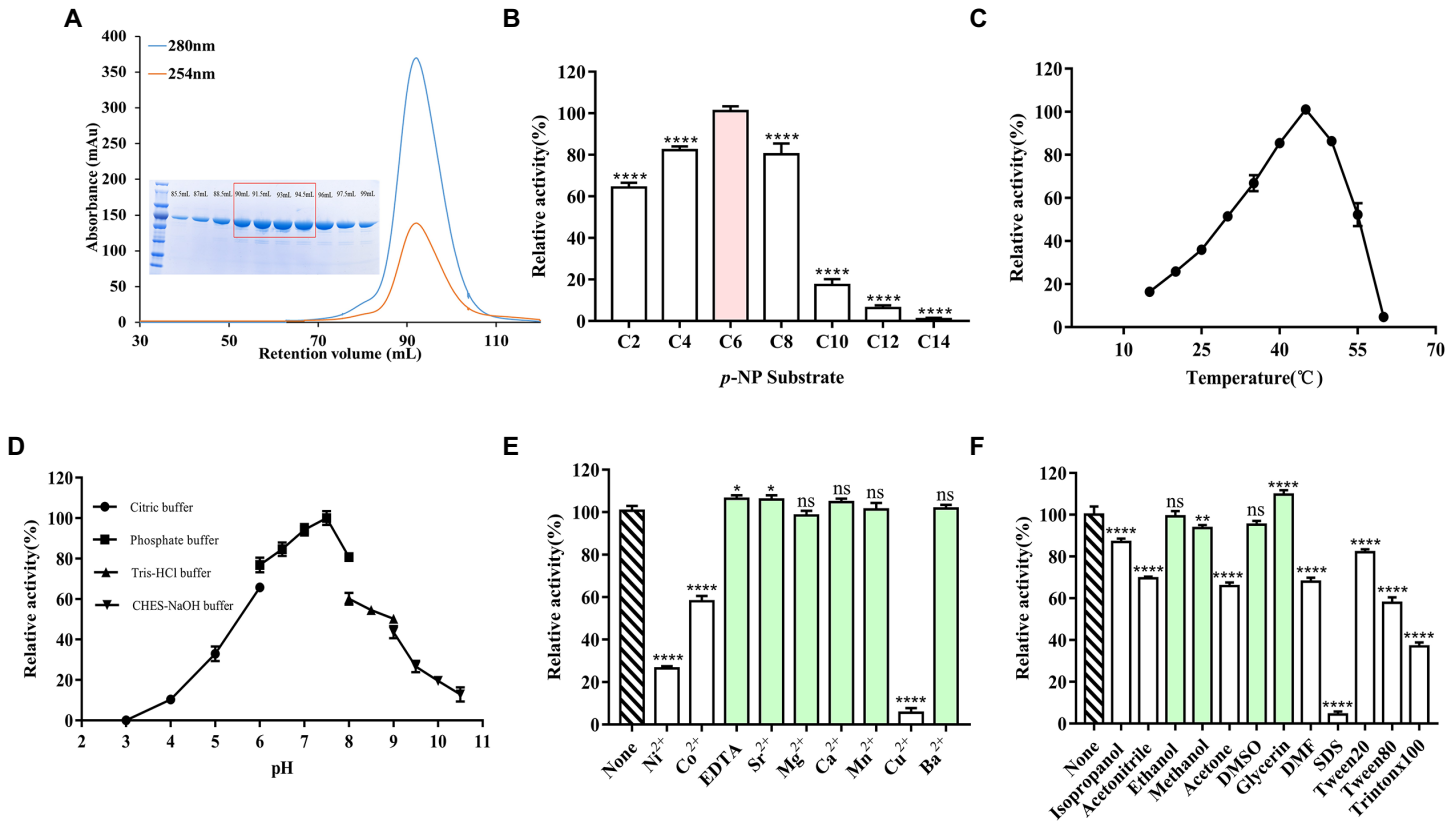
Catalytical features of E93 with *p*-NP esters as substrates. **(A)** Gel filtration chromatography and SDS-PAGE results of E93. High-purity protein samples were collected for crystallization (in the red box). **(B)** Enzymatic activities to substrates with various fatty acid chain of *p*-Nitrophenyl (*p*-NP) esters. The catalytic activity of E93 to *p*-NP hexanoate (C6) was 100%. **(C)** Effects of temperature on enzyme activities. Enzymatic activities were measured in a series of temperatures. The activity obtained at 45°C was taken as 100%. **(D)** Effects of different pH on enzyme activities. Enzymatic activities were determined under a series of pH. The activity obtained at pH 7.5 was taken as 100%. **(E)** Tolerance of ions. The reaction without additional ions was used as a control. The C6 substrate was used for measurements. **(F)** Effects of organic solvents on the enzymatic activity. The reaction without any organic solvent was used as a control. The solvents with no inhibition effect on the enzyme activity were labeled with light green color in panels **(E,F)**. The data showed in panels **(B–F)** is from triplicate experiments (mean ± SD). *p < 0.05, **p < 0.01, ***p < 0.001, ****p < 0.0001, and ns, not significant.

E93 with his-tag at the N-terminus was expressed in *Escherichia coli* BL21 (DE3). The molecular weight of E93 was 57 kDa (Figure 2A). The catalytic assay with a series of *p*-NP esters indicated a substrate preference. E93 specifically hydrolyzed *p*-NP ester substrates with short fatty acid chains. The substrates with fatty acid chains longer than 10 carbon atoms were poorly hydrolyzed (with a catalytic activity of 20% or less, compared with optimal the *p*-NP substrate, *p*-NP hexanoate; Figure 2B). The optimum temperature was 45°C. The catalytic capability attenuated when the temperature was higher than 55°C or lower than 30°C (Figure 2C). The catalytic activity was retained under a pH between 6.0 and 8.5. The optimal pH was 7.5 (Figure 2D).

Incubation with EDTA did not appreciably change the catalytic activity (105% compared to the activity in standard conditions), indicating that the catalytic activity of E93 was independent of metal cations. More than 70 and 90% loss of activity, respectively, indicated significant inhibition of Ni^2+^ and Cu^2+^ to the catalytic activity of E93. E93 was slightly inhibited by Co^2+^ and 60% activity was retained. In addition, cations, including Sr^2+^ (104%), Mg^2+^ (98%), Ca^2+^ (103%), Mn^2+^ (100%), and Ba^2+^ (99%), were not detrimental to the catalytic activity of E93 (Figure 2E).

The catalytic assay with the treatment of organic reagents indicated that the esterase activity of E93 was not influenced by glycerin, ethanol, methanol, or DMSO. E93 can also be tolerant to isopropanol, acetonitrile, acetone, DMF, Tween 20, and Tween 80, with 50–80% of activity. The SDS and Triton X100 surfactants significantly inhibited the E93 esterase activity (Figure 2F).

### 2.3. Crystal structure of E93

**Figure 3:**
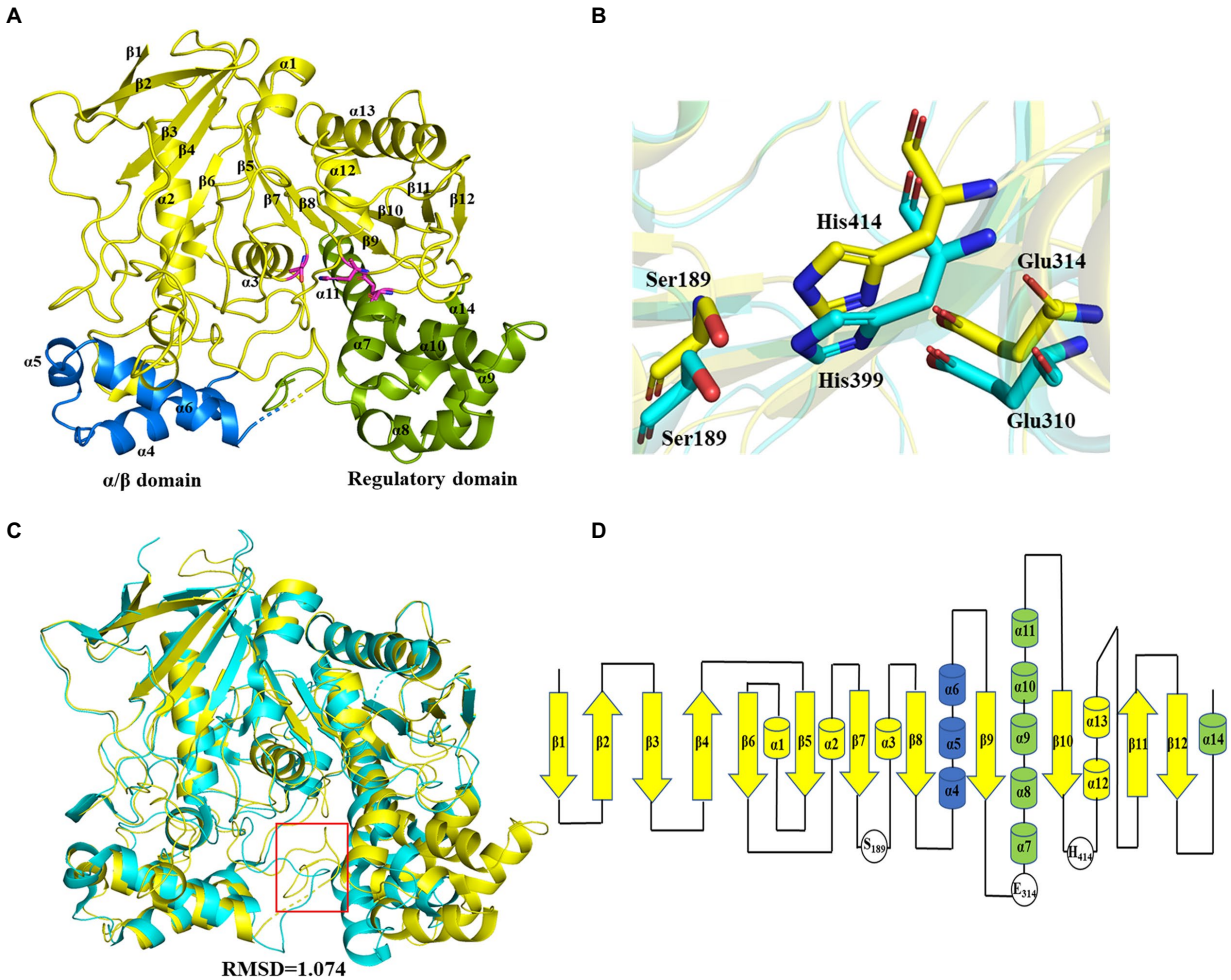
Crystal structure of E93 and structure comparison with microbial family VII carboxylesterases. **(A)** Global structure of E93. The structure contains three domains, including the catalytical domain (yellow), α/β domain (blue), and regulatory domain (green). **(B)** Conformational comparison of the catalytic triad of E93 (S189, E314, and H414; yellow) with that of pnbCE (S189, E310, H399; cyan). **(C)** Crystal structure comparison of E93 (yellow, PDB code: 7X8L) with pnbCE (cyan, PDB code: 1QE3). E93 and pnbCE are structurally homologous proteins. The RMSD value of E93 and pnbCE is 1.074 (Cα 328). The unique loop (271aa-277aa in E93 and 268aa-274aa in pnbCE) was shown in red box. **(D)** Structural topology of E93. The color scheme is consistent with that of Figure 2A.

The crystal structure of wild-type E93 with a resolution of 1.77 Å (PDB entry: 7X8L) was constructed by molecular replacement using rCE (PDB entry: 1K4Y) as a model (Supplementary Table S1). A total number of 510 amino acids were precisely placed in the electron density map. The structure contained 14 α-helices and 12 β-sheets. E93 presented the classical structural features of family VII esterases, where the whole structure consisted of three domains: the catalytic domain, α/β domain, and regulatory domain (Figure 3A). The catalytic domain was composed of β1–β12, α1–α3, and α12–α13, exhibiting a typical α/β hydrolase structure, where the 12 β-sheets were surrounded by five α-helices (Figures 3A,D) and the catalytic triad consisted of S189, E314, and H414 (Figure 3B; Supplementary Figure S2). The α/β domain was composed of the α4-α6; this domain was structurally conserved across family VII carboxylesterases (Figure 3C). The regulatory domain was composed of α7-α11, and α14. This domain was varied among members in Family VII carboxylesterases (Figure 3C).

### 2.4. E93 pocket comparison with related bacterial proteins

**Figure 4:**
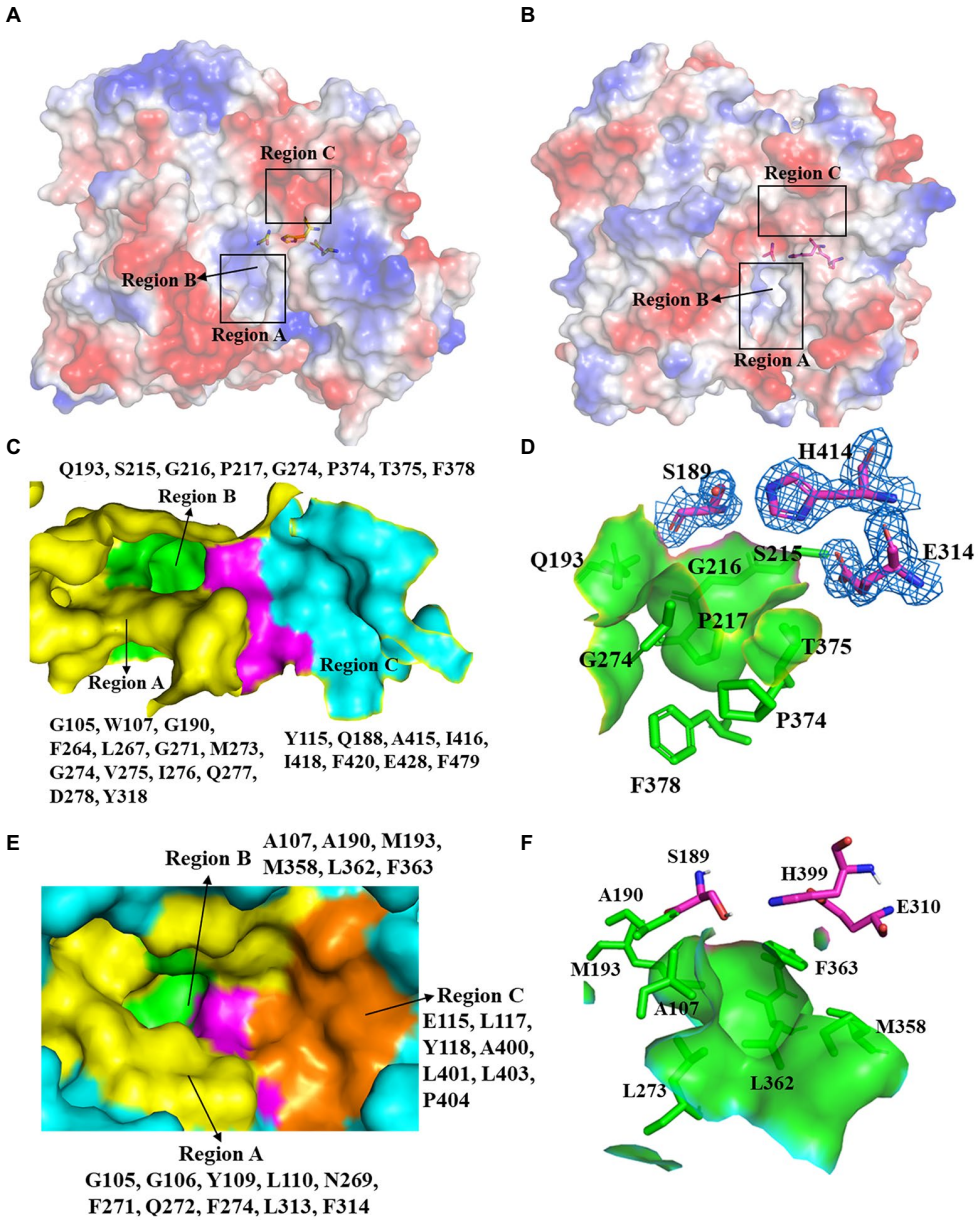
Comparison of the catalytic pockets of E93 and pnbCE. **(A,B)** The electrostatic surfaces of E93 **(A)** and pnbCE **(B)**. Sticks show catalytic triad. The positive charged regions are shown in blue and the negative charged regions are shown in red. Regions A–C are specifically noted. **(C)** The detailed exhibition of catalytic pocket of E93 and the amino acid composition of each region. The magenta area indicates the catalytic triad. **(D)** Specific features of the Region B in E93. The amino acids that constitute Region B are shown as green sticks. The catalytic triad of E93 is precisely immerged in an electronic density map contoured to 1.0 σ at the *2Fo-Fc* map closing to the Region B. **(E)** The detailed exhibition of catalytic pocket of pnbCE and the amino acid composition of each region. The magenta area indicates the catalytic triad. Amino acids that comprise different regions are shown. **(F)** Specific features of the Region B in pnbCE. The catalytic triad is shown with magenta stick. The amino acids that constitute Region B are shown with green sticks.

pnbCE (PDB entry: 1QE3) is a family VII carboxylesterases which can hydrolyze CPT11 (Wierdl et al., 2004) The RMSD value of E93 and pnbCE was 1.074 (Cα: 328; Figure 3C). Structural comparison showed that the catalytic triad of E93 (S189, E314, H414) had the same conformation as that of pnbCE (S189, E310, H399; Figure 3B). They were highly conserved throughout the family VII carboxylesterase and essential for catalytic reactions (Supplementary Figure S2). The mutation S189A disabled E93 from the hydrolysis of all ester substrates (Supplementary Tables S2–S4). A comparison of the catalytic pockets of E93 and pnbCE indicated that the catalytic pocket of both enzymes formed an open and long cleavage (Figures 4A,B). The overall charge properties of the catalytic pocket in E93 and pnbCE were similar. The catalytic triad was located in a negatively charged region, and the overall pocket had neutral and positively charged properties (Figures 4A,B).

**Figure 5:**
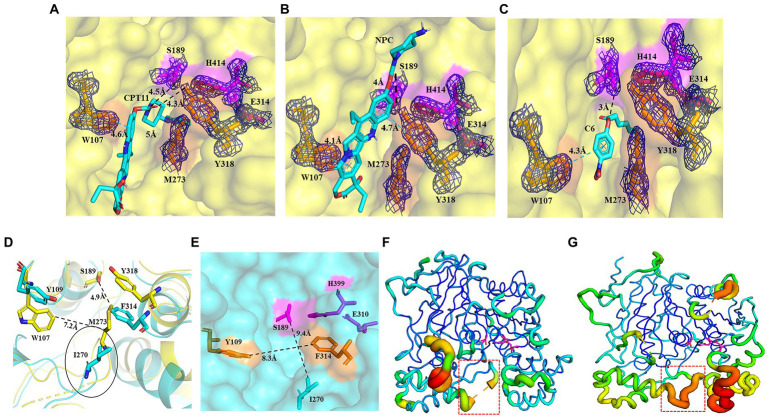
Binding patterns of E93 with different substrates in the Region A of catalytic pocket, simulated by AutoDock. **(A–C)** The docking of CPT11 **(A)**, NPC **(B)**, and *p*-NP hexanoate (C6) **(C)** into the catalytic pocket of E93. Magenta sticks represent the catalytic triad. The large-sized amino acids (Orange) involved in forming the tunnel structure of the Region A in E93 are shown with orange sticks. The interactions of E93_W107 and E93_Y318 with the alcoholic group of CPT11, NPC, and C6 are shown. The interaction between the catalytic residue S189 and the carbonyl carbon of substrate is also demonstrated. The black dash line denotes hydrogen bond, and the blue dash line denotes π–π interaction. The electronic density map is contoured to 1.0 σ at the *2Fo-Fc* map. **(D)** Structural superposition of the large amino acids within Region A of E93 (yellow) and the corresponding amino acids of pnbCE (cyan). The Region A of E93 has a width of 7.2 Å. The Special loop structure with significant conformational difference is specifically labeled. The distance between catalytic S189 and M273 in the special loop is 4.9 Å. **(E)** The large-sized amino acids involved in forming the tunnel structure of the Region A in pnbCE are shown with orange sticks. The catalytic triad of pnbCE is highlighted by the magenta sticks. The Region A of pnbCE has a width of 8.3 Å. The distance between pnbCE_I270, corresponding to E93_M273, and the catalytic resideue, S189 in pnbCE, is 9.4 Å. The dash line in panels **(D,E)** was used to show the distance between two amino acids. **(F,G)** B-factor analysis of E93 **(F)** and pnbCE **(G)**. The thickness of coil represents the flexibility of the structure. The thicker the coil is, the higher flexibility the structure is. The catalytic triad residues of E93 and pnbCE are shown as sticks. The loop with high flexibility in catalytic center of two enzymes is labeled with red box.

The catalytic pocket of E93 can be divided into three regions (Region A–C) by the catalytic triad (Figures 4A,C). Carboxylesterase pnbCE showed a similar pocket pattern (Figures 4B,E). The Region A in E93 was composed of G105, W107, G190, F264, L267, G271, M273, G274, V275, I276, Q277, D278, and Y318 (Figure 4C). Two residues, W107 and Y318, were directly involved in the formation and determined the relatively narrow tunnel of the Region A in E93 (Figures 5A–D). The corresponding residues in pnbCE were Y109 and F314 (Figures 5D,E). A loop with high flexibility was identified in both E93 (residues 271–277) and pnbCE (residues 268–274) (Figures 5F,G). This loop is located at the core catalytic area in the pocket and was involved in the formation of Region A (Figure 5D). A residue, M273, on the flexible loop in E93 was also involved in the formation of the tunnel in Region A (Figure 5D). Compared with pnbCE, the loop of E93 was located closer to the inside of the pocket, resulting in a narrower size in the tunnel of the pocket (with a distance of 7.2 Å) compared with that of pnbCE (8.3 Å) (Figures 5D,E). This difference also led to the shorter distance between the long side chain of M273 and the catalytic residue S189 in E93 (4.9 Å) compared with the distance between the corresponding residue in pnbCE, I270, and its catalytic center, S189 (9.4 Å; Figures 5D,E).

The Region B was a region near the catalytic triad, and it can be identified in both E93 and pnbCE (Figures 4C,E). The Region B in E93 was enriched with polar amino acids and was composed of Q193, S215, G216, P217, G274, P374, T375, and F378 (Figure 4D). This region in pnbCE was composed of nonpolar residues (Figure 4F).

Region C in E93 was composed of Y115, Q188, A415, I416, I418, F420, E428, and F479. (Figure 4C). Correspondingly, Region C in pnbCE comprised E115, L117, Y118, A400, L401, L403, and P404 (Figure 4E). Similar to E93, Region C of pnbCE was enriched with non-polar amino acids.

### 2.5. AutoDock of E93 with CPT11, NPC, and *p*-NP ester substrate

**Figure 6:**
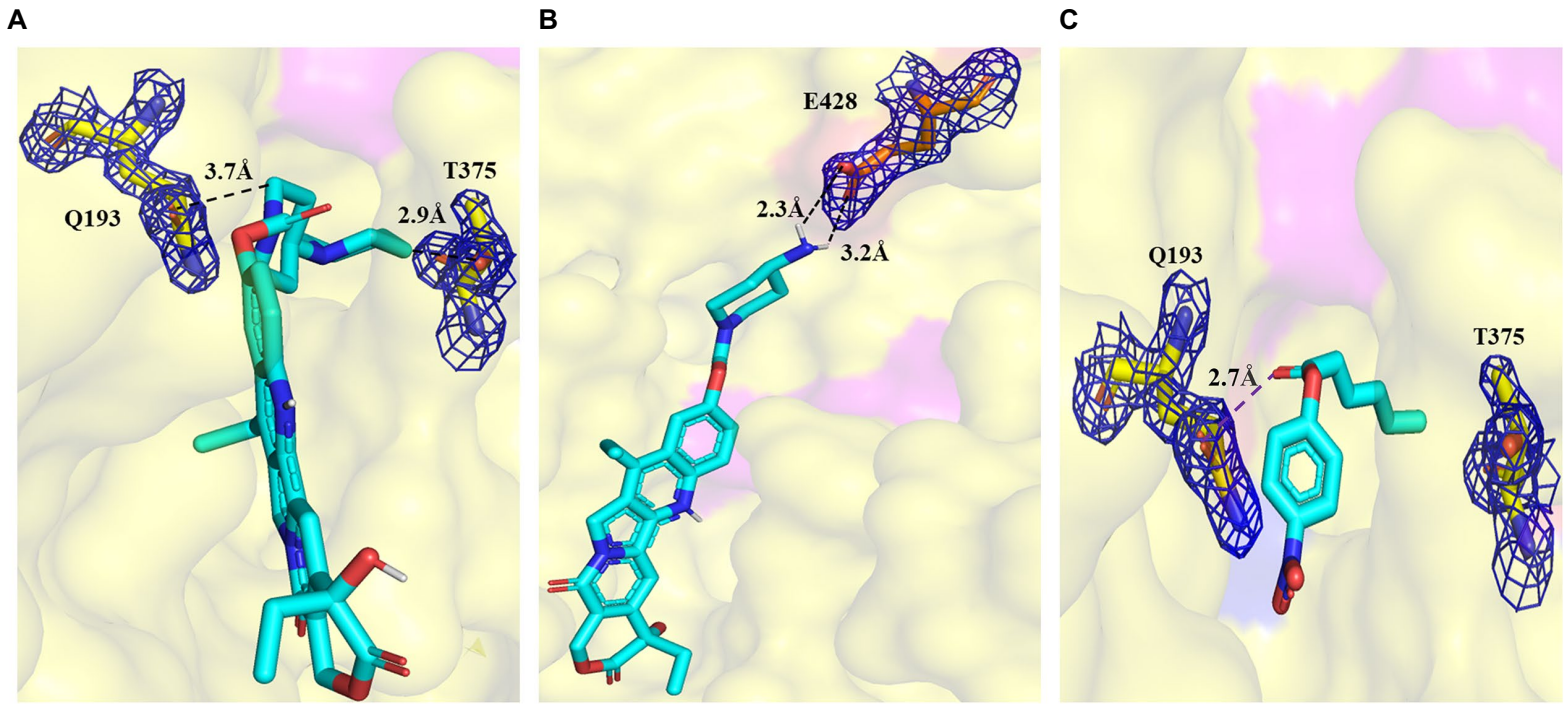
Details of the binding of CPT11 and C6 in Region B, and NPC in Region C. **(A)** Interaction of Q193 and T375 with the 4PP group of CPT11 in Region B. The dashed lines illustrate hydrogen bonds. **(B)** Interaction of E428 with the acyl groups of NPC within Region C. Dashed lines illustrate hydrogen bonds. **(C)** The relative locations of *p*-NP hexanoate (C6), Q193, and T375 in Region B. The dashed lines illustrate interaction. The electronic density map is contoured to 1.0 σ at the *2Fo-Fc* map.

To better understand the binding of different substrates within the E93 pocket, a small-sized ester substrate (*p*-NP hexanoate (C6)) and large anticancer prodrug substrates, CPT11 and NPC, were used as ligands for docking into the pocket of E93. The AutoDock result showed that the alcohol group of both CPT11 and NPC were docked at the Region A of the pocket, where the large-sized residues of E93, W107, M273, and Y318 were involved in the binding with the substrates (Figures 5A–C). The acyl group of CPT11 and NPC, however, bound to different regions of the catalytic pocket. The acyl group of CPT11 is bound to the polar Region B of E93 (Figures 5A, 6A). Two residues in Region B, Q193, and T375, were close enough to generate interaction with the acyl group of CPT11 (3.7 Å and 2.9 Å, respectively) (Figure 6A). The acyl group of NPC was docked into the Region C of E93 (Figures 5B, 6B). As the only acidic residue in non-polar Region C, the E428 might have contact with NPC (Figure 6B).

The alcohol group of the C6 substrate was docked at the Region A, while the fatty acid acyl group was docked at the Region B (Figures 5C, 6C). Two residues in Region A, W107 and M273, and two residues in Region B, Q193 and T375 might have contact with C6 substrate, maintaining the short distance between the ester bond of C6 substrate and the catalytic triad (S189) of E93 (Figures 5C, 6C).

### 2.6. Prodrug hydrolysis of E93 and its mutants

**Figure 7:**
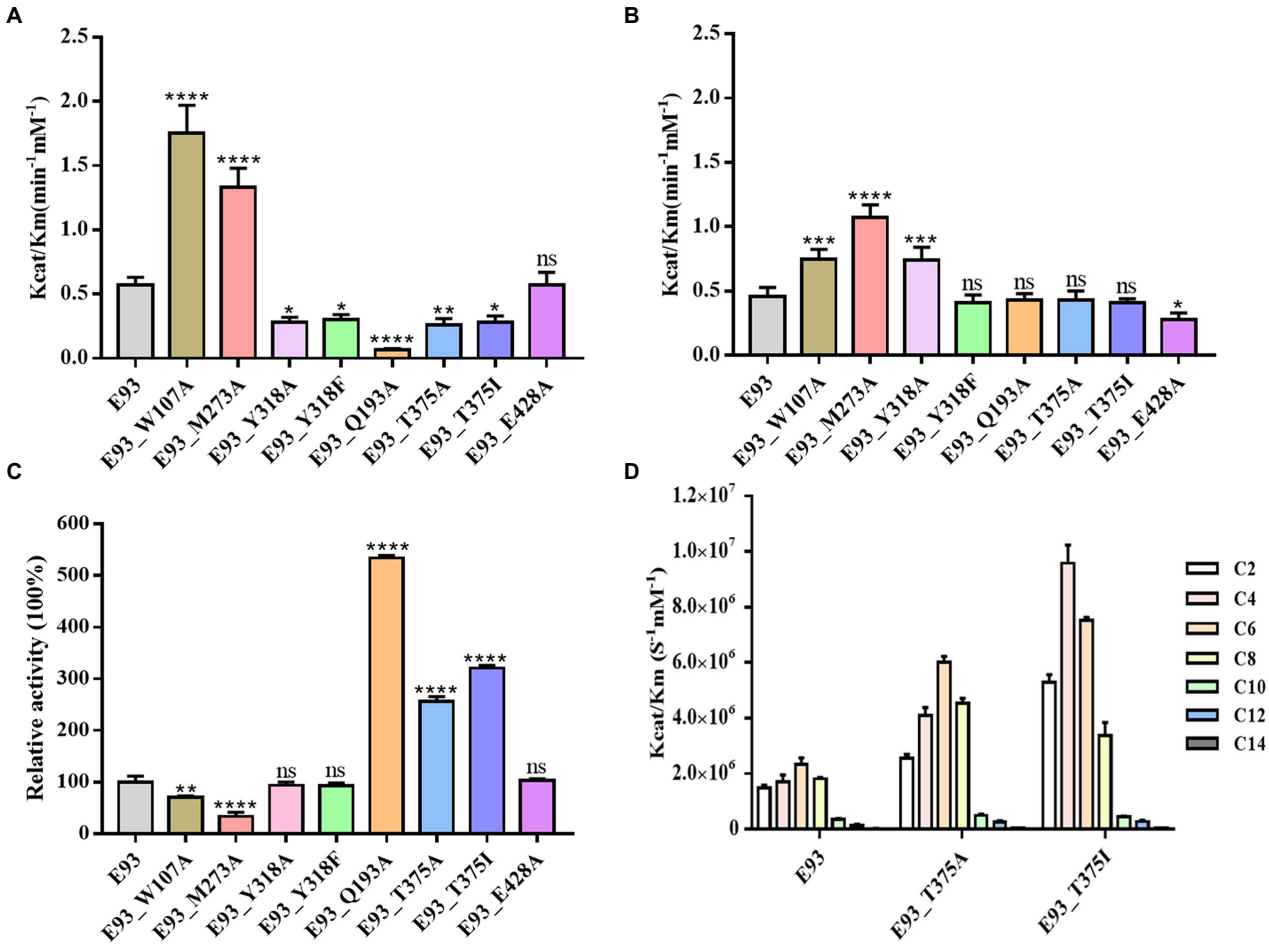
Mutagenesis analysis of key residues involved in hydrolyzing CPT11, NPC, and *p*-NP hexanoate (C6). Comparison of the hydrolytic activities of each mutant for CPT11 **(A)**, NPC **(B)** and *p*-NP hexanoate (C6) **(C)**. The activity of wild-type E93 was taken as 100% for panel **(C)**. **(D)** Catalytic activity of wild-type E93, E93_T375A, and E93_T375I on *p*-NP ester substrate hydrolysis. The data showed in panels **(A–D)** is from triplicate experiments (mean ± SD). *p < 0.05, **p < 0.01, ***p < 0.001, ****p < 0.0001, and ns, not significant.

The results of the activity assay indicated that E93 was capable of prodrug CPT11 activation and the production of SN38. The mutation of W107, M273, and Y318 in Region A had effects on CPT11 and NPC substrates. The catalytic activity of the W107A mutant was 3-and 1.5-fold higher to CPT11 and NPC substrates, respectively. Compared to the wild-type E93 (Figures 7A,B; Supplementary Tables S2, S3). The M273A mutant had 2.3-fold increase in hydrolytic activity to both CPT11 and NPC substrates (Figures 7A,B; Supplementary Tables S2, S3). The Y318 showed opposite effects on the hydrolysis of CPT11 and NPC. Both Y318A and Y318F mutants exhibited reduced catalytic activity for the CPT11 substrate by approximately 51 and 47%, respectively (Figure 7A; Supplementary Table S2). In contrast, Y318A and Y318F showed increased catalytic activity or no significant effects on the NPC substrate hydrolysis (1.6-fold increase to Y318A mutant and no significant change to Y318F mutant compared with wild-type E93) (Figure 7B; Supplementary Table S3).

Mutation in Region B gave a significant impact on CPT11 hydrolysis but not on NPC hydrolysis (Figures 7A,B; Supplementary Tables S2, S3). The hydrolytic activities of Q193A, T375A, and T375I mutants on CPT11 were reduced by 88, 54, and 51%, respectively (Figure 7A). In addition, a significant reduction in CPT11 substrate binding affinity was also observed in Q193A mutant (Supplementary Table S2).

In region C, E93_E428A mutant lost 40% of its hydrolytic activity and had a higher Km value for NPC substrate without affecting the activity for the CPT11 substrate (Figures 7A,B; Supplementary Tables S2, S3). The result of enzymatic assays is consistent with the AutoDock prediction, indicating that the CPT11 and NPC had different substrate-enzyme binding patterns during the hydrolysis.

### 2.7. *p*-NP ester hydrolysis of E93 and its mutants

The effects of the mutations at six residues (W107, M273, and Y318 from Region A; Q193 and T375 from Region B; E428 from Region C) to *p*-NP substrates hydrolysis was different from that to CPT11 and NPC. The W107A and M273A mutation in Region A impaired the catalytic activity to C6 substrate hydrolysis (30% loss of activity to W107A and 67% loss of activity to M273A) (Figure 7C; Supplementary Table S4). Both mutants also showed higher *K*m than wild-type E93 (1.2 and 2.2 times higher, respectively) (Figure 7C; Supplementary Table S4). Neither the Y318F nor Y318A mutation in the same region give a significant impact on C6 substrate hydrolysis. (Figure 7C; Supplementary Table S4).

Mutations at Q193 and T375 in Region B affected the catalytic activity in hydrolyzing *p*-NP substrates. The Q193A mutant significantly enhanced the catalytic activity of E93 by 5-fold with a 45% decrease in *K*m values (Figure 7C; Supplementary Table S4) while T375A mutant enhanced catalytic activity by 2.6-fold. The T375I mutant not only showed an increase of catalytic activity to C4 and C6 (6-and 3.2-times, respectively) substrates, but altered *p*-NP substrate preference. The optimal substrate was changed from *p*-NP hexanoate (C6) to *p*-NP butyrate (C4) substrate (Figure 7D). Furthermore, the *K*m values of T375I for C4 and C6 hydrolysis were lower than those of wild-type E93 (Supplementary Tables S4, S5). The mutation E428A at Region C did not change the hydrolytic activity of E93 toward small-sized substrates (Figure 7C). The result of the enzymatic assay on *p*-NP ester substrate verified AutoDock prediction.

## 3. Discussion

CPT11 is a popular broad-spectrum anticancer drug. The functional anticancer drug SN38 is released by carboxylesterase-mediated hydrolysis of ester bonds between SN38 and 4PP (Palakurthi, 2015). The pnbCE, identified from soil bacteria, is a bacterial Family VII carboxylesterases that can hydrolyze CPT11 (Wierdl et al., 2004). However, knowledge concerning enzyme-substrate specificity and prodrug-enzyme interactions in microbial family VII carboxylesterases is rare. In addition, the catalysis to NPC substrate was exclusive from previous studies. To our knowledge, bacterial esterases with NPC hydrolysis activity have also not been reported yet.

In our previous work, a group of carboxylesterases was identified from marine bacteria. The CPT11 and NPC were used as potential substrates to screen the prodrug activation of the enzymes. The bacterial family VII carboxylesterase E93 was identified to be capable of both CPT11 and NPC hydrolysis. It was the first time to experimentally verified the catalytic capability to both CPT11 and NPC substrates in bacterial carboxylesterase. We thought the E93 was an appropriate subject to uncover the catalytic mechanism of carboxylesterase on CPT11 and NPC hydrolysis. The structure-based, as well as molecular docking approaches were used to analyze the key structures and residues of E93 on CPT11 and NPC hydrolysis.

### 3.1. The region a of E93 was required for the binding with alcohol group of CPT11 and NPC substrates

The CPT11 and NPC shared the same alcohol group, SN38 (Figure 1). This polyaromatic structure required a pocket region with large space for accommodation. AutoDock analysis located the alcohol group of both substrates in Region A, and three large-sized residues, W107, M273, and Y318, might have interaction with the substrates (Figures 5A,B). The W107A mutation significantly improved the catalytic activity to both CPT11 and NPC (Figures 7A,B), indicating that the steric hindrance from the side chain of W107 was not beneficial for the binding with CPT11 and NPC.

A loop with high structural flexibility was identified at both pnbCE and the region A of E93 (Figures 3C, 5F,G). The M273 was located at this loop, and the distance between M273 and one residues of catalytic triads, S189, might affect the binding of substrates (Figure 5D). In the E93, this loop was closer to the catalytic triads compared with pnbCE, leading to the shorter distance (4.9 Å) between M273 and S198 (Figure 5D). This distance might be adequate to affect the binding of the enzyme to CPT11 and NPC. By removing the long side-chain of the residue, the M273A mutation significantly improved the catalytic activity to both substrates (Figures 7A,B), emphasizing that the steric hinderance as well as the space between M273 and S189 might determine the substrate catalytic activity as well as substrate specificity.

It is worth noting that the Y318A mutation gave different impact on CPT11 and NPC activation. The mutation significantly enhanced the catalytic activity to NPC activation but attenuated the activity to CPT11 activation (Figures 7A,B). The AutoDock results indicated that the location of ester in the catalytic pocket was different for CPT11 and NPC (Figures 5A,B). The carbonyl oxygen of CPT11 was close to the side chain of Y318. A possible hydrogen interaction might be established between CPT11 and Y318, and contributed to the binding of CPT11 to the catalytic pocket of E93 (Figures 5A, 7A; Supplementary Table S2). However, such interaction was impossible to be established to NPC. Instead of carbonyl oxygen, the benzene ring next to ester bond of NPC was close to the aromatic side-chain of Y318 (Figure 5B). The steric hindrance, instead of a possible hydrogen bond interaction, might gave impact on the substrate-enzyme interaction to NPC. This hypothesis was partly verified by the result of Y318A enzymatic assay. The removal of side chain of Y318A greatly improved the catalytic activity to NPC substrate as well as the affinity between NPC and E93 (Figure 7B; Supplementary Table S3). To furtherly verify the hypothesis, the Y318F mutant was designed. The Y318F eliminated the hydroxyl which can establish possible hydrogen bond to substrate, without changing the size of side chain. Different from Y318A, the Y318F attenuated the catalytic activity to CPT11 activation (Figure 7A). However, the catalytic activity to NPC activation was not influenced by the mutation (Figure 7B). The hydrogen bond between CPT11 and Y318 facilitated the activation of CPT11 by E93, while the steric hinderance was detrimental for the catalytic activity to NPC activation. The binding feature of the alcohol group of CPT11 and NPC was different even if both were binding in the same region of the catalytic pocket.

### 3.2. The acyl group of CPT11 and NPC bound to different region of E93 catalytic pocket

The Autodock analysis located the acyl group of CPT11 and NPC in different region of E93 catalytic pocket (Figures 6A,B). The acyl group of CPT11 was docked at the region B of catalytic pocket where the region was composed of polar amino acids (Figure 4D). The acyl group of NPC was docked at the region C, a non-polar region of the pocket (Figures 4C, 6B). The 4-piperidino-piperidine acyl group of CPT11 had stronger polarity than the mono-piperidine acyl group of NPC. Such difference also determined the different binding feature of alcohol group of CPT11 and NPC (Figures 6A,B). In order to understand the role of polar interaction to the binding of the pocket with prodrug substrates, the main polar residues in region B, Q193 and T375, were mutated. The replacement of polar residues with non-polar amino acid alanine greatly reduced the catalytic activity of CPT11 activation as well as enzyme-CPT11 binding affinity (Figure 7A; Supplementary Table S2). The similar result was also obtained to T375I mutant. The analysis revealed that the polar interaction established among the acyl group of CPT11 and the polar residues in region B contributed to the E93-CPT11 interaction. The mutations in region B did not gave significant effect to the catalytic activity of E93 to NPC hydrolysis (Figure 7B; Supplementary Table S3), confirming that the region B was not directly involved in NPC binding.

On the other hand, a mutation in region C was developed to analyze possible interaction between the enzyme and the acyl group of the substrates. The E428 was the only acidic amino acid in non-polar region C. The AutoDock analysis showed a possible hydrogen bond interaction in between the acyl group of NPC and the side chain of E428 (Figure 6B). The E428A mutation significantly reduced the activity of E93 to NPC hydrolysis as well as the binding affinity between the enzyme and NPC (Figure 7B; Supplementary Table S3), implying the importance of E428 to the NPC hydrolysis. The E428A mutation did not change the activity of E93 to CPT11 activation. Taken together, the interaction between the catalytical pocket and the acyl group played key role for activation of CPT11 and NPC. A small structural difference in acyl group also lead to the significant difference in enzyme-substrate binding of CPT11 and NPC.

### 3.3. The alcohol group of *p*-NP substrate bound to the region a of E93 catalytic pocket

The Autodock analysis docked the optimal *p*-NP substrate, C6 substrate, at the region A and region B of the catalytic pocket (Figures 5C, 6C). The alcohol group of C6 substrate was docked at the region A. Different from CPT11 and NPC, the alcohol group of C6 contained only one benzene ring. The small size of alcohol group might lead to different binding features of C6 substrates, compared with CPT11 and NPC. The W107A and M273A mutants significantly reduced the activity of E93 to C6 substrate hydrolysis (Figure 7C). The binding affinity of E93 and C6 substrate was also impaired (Supplementary Table S4). The effects of mutation on catalytic activity to C6 substrate was different from that of CPT11 and NPC substrates (Figures 7A–C). Structure analysis indicated a possible π-π interaction between the aromatic side-chain of W107 and the alcohol group of the C6 substrate (Figure 5C). The side-chain of M273 established a hydrophobic interaction with the C6 substrate, facilitating the catalysis of E93 to C6 subtrate (Figure 5C). The data demonstrated that the large-sized amino acids, W107 and M273, which were unfavorable for the CPT11 and NPC hydrolysis, contributed to the binding of *p*-NP substrate by offering a hydrophobic environment at the alcohol group of the substrate.

### 3.4. The acyl group of *p*-NP substrate bound to the region B of E93 catalytic pocket

The acyl group of C6 substrate was docked at the region B of the catalytic pocket by AutoDock analysis (Figure 6C). Due to the chemical property of the acyl group of C6 substrate and the amino acids at the region B, the polarity of region B might limit the binding affinity of non-polar fatty acid chain of the acyl group of C6 substrate. The replacement of polar amino acids Q193 and T375 with non-polar amino acids improved both the activity and binding affinity with C6 substrate (Figure 7C; Supplementary Table S4), verifying the role of polarity of region B to the *p*-NP substrate catalysis. In addition, the residue T375, which was located at the bottom of region B played a key role on *p*-NP substrate specificity (Figures 4D, 7C,D). The optimal *p*-NP substrate became C4 substrate in T375I mutant (Figure 7D; Supplementary Table S5), while the catalytic activity of E93 to C8 substrate had a great increase in T375A mutant.

The study identified a bacterial carboxylesterase, E93, which can active both the prodrug CPT11 and its metabolic derivative NPC. With the structural and enzymatic analysis of this newly identified enzyme, the catalytic mechanism of CPT11 and NPC hydrolysis was discussed. The analysis of E93 helped the deep understanding of prodrug activation, improving the application of bacterial carboxylesterase in medical use.

## 4. Materials and methods

### 4.1. Bacterial cultivation and recombinant plasmid construction

*A. indicus* DSM18604^T^ was obtained from the China General Microbiological Culture Collection (CGMCC). Cells were cultivated in Marine 2,216 medium (Difco, pH 7.5) at 30°C. *E. coli* BL21 (DE3) was cultivated in LB medium (pH 7.0) at 37°C. The genomic DNA of *A. indicus* was extracted using a bacterial genomic DNA purification kit (Thermo Scientific, Waltham, MA, USA). The WT *e93* gene was amplified by polymerase chain reaction (PCR) with the primers listed in the supporting information (Supplementary Table S6). The pSMT3 SUMO-tag system was used in this study. The SUMO-tag contains six histidines at the N-terminal region, which can bind with the Ni-NTA affinity column. The PCR product and backbone of pSMT3 plasmid were digested by *Nde I* and *Hind III* (New England Biolabs, United States) at 37°C for 1 h, and followed by kit-based DNA purification (Thermo Fisher Scientific, United States) and T4 DNA ligase-mediated ligation (New England Biolabs, United States). The single amino acid mutation was introduced *via* the kit-based mutation system (Fast Mutagenesis System, TransGen Biotech) with the WT gene *e93* as a template. The primers required for construction of mutation was shown in the Supplementary Table S6. The recombinant plasmid was transferred into *E. coli* BL21 (DE3) competent cells using a heat-shock approach. Recombinant *E. coli* BL21 (DE3) clones were selected from LB plates containing kanamycin (50 μg/ml). The inserted DNA fragments were confirmed by PCR and DNA sequencing.

### 4.2. Protein production and purification

The recombinant *E. coli* BL21 (DE3) strain was cultivated at 37°C in LB medium with kanamycin (50 μg/ml). The production of wild-type E93 and its mutants were induced by 0.5 mM isopropyl-β-D-thiogalactopyranoside (IPTG) for 16 h at 20°C. The cells were harvested and disrupted using an ultrasonic homogenizer (Scientz-IID, China). The lysates were sequentially purified by Ni-NTA affinity chromatography (Thermo Scientific) using imidazole step elution. The protein concentration was determined using the Bradford method.

### 4.3. Docking simulation

Docking simulations were performed using AutoDock 4.0 (Jiang et al., 2008). AutoDock tools initialized the ligands by adding gasteiger charges, merging nonpolar hydrogen bonds, and setting rotatable bonds. The ligands were rewritten into PDBQT format, which can be read by AutoDock. AutoDock Tools were used to add polar hydrogen to the entire receptor. The grid box was set to contain the entire receptor region. The receptor output was also saved in PDBQT format. AutoDock was set with the macromolecule held fixed and the ligands flexible. Determination of the location of the ligand binding pocket by catalytic triad. The grid box was set to cover all residues within 10 Å radius sphere around the catalytic Ser189 residue thus compassing the entire catalytic cavity. The resolution of the grid was 60 × 60 × 60 points with a grid spacing of 0.375 Å. Each substrate was docked into this grid using the Lamarckian algorithm, as implemented in AutoDock. The flexible bonds of the ligand were automatically recognized and allowed to rotate. The genetic-based algorithm ran 20 simulations per substrate with 2,000,000 energy evaluations and a maximum of 27,000 generations. The crossover rate was increased to 0.8, and the number of individuals in each population was 150. All other parameters were set at the AutoDock default settings (Jiang et al., 2008). The docking results from each calculation were clustered based on root-mean-square deviation (rmsd) (solutions differing by less than 2.0 Å) between the cartesian coordinates of the atoms and were ranked based on the predicted binding free energy. The docking structure with low free energy and reasonable distance between catalytic triad and ester bond of substrate was selected.

### 4.4. Biochemical characterization of E93 and its mutants

The substrates *p*-nitrophenyl (NP) acetate (C2), *p*-NP butyrate (C4), *p*-NP caprylate (C8), *p*-NP decanoate (C10), *p*-NP laurate (C12), *p*-NP myristate (C14), and *p*-NP palmitate (C16) were purchased from Sigma-Aldrich (St. Louis, MO, USA), and *p*-NP hexanoate (C6) was purchased from TCI (Japan). The standard reaction was conducted with 50–100 μg of purified E93 or its mutants (due to the discrepancies in the activity among the wild-type enzyme and its mutants) in a 1.0 ml mixture containing 100 mM Tris–HCl (pH 7.5) buffer and 1 mM *p*-NP esters. The enzyme activity was determined at 30°C at 405 nm using DU800 UV/Visible spectrophotometer (Beckman Coulter, Brea, CA, USA). The absorbance values were measured every 15 s for 2 min. The rate of change was used for the subsequent determination of enzyme activity. The standard curve of the *p*-NP product was obtained at a wavelength of 405 nm before every measurement. All experiments were performed in triplicate and corrected for substrate autohydrolysis. The kinetic parameters were obtained using *p*-NP butyrate as a substrate at different concentrations ranging from 0.02 to 3.50 mM under the optimal conditions (pH 7.5, 45°C, and Tris–HCl buffer). The kinetic parameters were calculated by analyzing the slopes of the Michaelis–Menten equation using GraphPad Software (GraphPad Inc., San Diego, CA, USA). The optimal pH test was performed in the same reaction system with potassium phosphate buffer (pH 5.0–7.5), Tris–HCl buffer (pH 7.5–9.0), and CHES buffer (pH 9.0–10.5). The effects of metal ions were measured at a final concentration of 10 mM. The effect of the chelating agent, ethylenediaminetetraacetic acid (EDTA), was determined at a final concentration of 10 mM. The effects of organic solvents were determined at a final concentration of 15% (v/v). The enzyme is co-incubated with organic solvents or metal ions in an optimal buffer for 5 min before the reaction starts.

### 4.5. HPLC assay for conversion of CPT11 and NPC to SN38

The reaction was conducted as previously described (Hough and Wilson, 2018) with minor modifications. In brief, freshly purified E93 and its mutants were incubated with different concentrations of CPT11 and NPC, between 5 μM and 200 μM in a final volume of 300 μl of 50 mM HEPES (pH 7.4) at 37°C for 10 h. As described previously, the kinetic parameters for the conversion of CPT11 and NPC were obtained from the HPLC separation of the substrate and product (Hosokawa, 2008). The reaction was terminated by adding one volume of 75 mM ammonium acetate (pH 4.0), vortexing, and centrifugation at 14,000× *g* for 5 min. An aliquot of the reaction mixture was injected into an Atlantis dC 18 chromatography column (4.6 mm × 50 mm) using an automatic injector (Amersham A-900) on an AKTA purifier. A step gradient elution was performed with 75 mM ammonium acetate (pH 4.0) and 0 and 25% acetonitrile to separate the substrate and product. The reaction mixture was eluted at 0.75 ml/min. The three compounds were detected based on UV absorbance at 266 nm and 375 nm. The peak areas of the products in the experimental samples were converted to the corresponding amounts of SN38 by plotting a standard calibration curve of the UV peak areas produced by the known amounts of SN38.

### 4.6. Crystallization and structure determination

The crystal of the E93 protein was obtained using a hanging drop method by mixing 1.0 μl of 10 mg/ml protein with 1.0 μl of reservoir solution at 18°C. The reservoir buffer contained 0.1 M Bis-Tris (pH 6.5) and 28% (v/v) PEG MME2000. The crystal samples were tested at the BL17U1 and BL19U1 beamlines at the National Center for Protein Sciences, China, and Shanghai Synchrotron Radiation Facility, China. The X-ray diffraction datasets were integrated, scaled, and merged using HKL3000. Phases were obtained by molecular replacement using Phaser with esterase rCE PDB coordinates (PDB no. 1K4Y) as the initial model. Crystallographic structure refinement was performed using Refmac5 in the CCP4 software suite (Hough and Wilson, 2018) and Phenix (Adams et al., 2010). The model was manually refined using Coot (Emsley and Cowtan, 2004). A structural similarity search was performed using the DALI server (Holm and Rosenstrom, 2010). All structures were visualized using the PyMOL software.^1^

### 4.7. Sequence alignment and 3D structure comparison

BLASTp analyzed the amino acid sequence of E93 against the UniProt database^2^ and the ESTHER database. Multiple sequence alignment was performed using ClustalX version 2.0. The corresponding phylogenetic tree was constructed using the neighbor-joining method in MEGA version 7.0 (Kumar et al., 2016). The crystal structure data of the family VII esterases were obtained from the RCSB Protein Data Bank.^3^ Structural comparisons were conducted using PyMOL software (Barber, 2021).

## Data availability statement

The datasets presented in this study can be found in online repositories. The names of the repository/repositories and accession number(s) can be found at: http://www.wwpdb.org/, 7X8L.

## Author contributions

YL, JL, and X-WX contributed to conceptualization, methodology, and writing-original draft. YL contributed to formal analysis, data curation, and visualization. YL, ZR, and ZL contributed to data curation and visualization. HC was contributed to visualization. JL and X-WX were responsible for supervision and project administration. X-WX was responsible for funding acquisition. All authors commented on previous versions of the manuscript, read and approved the final manuscript.

## Funding

This work was supported by the National Natural Science Foundation of China (32170005).

## Conflict of interest

The authors declare that the research was conducted in the absence of any commercial or financial relationships that could be construed as a potential conflict of interest.

## Publisher’s note

All claims expressed in this article are solely those of the authors and do not necessarily represent those of their affiliated organizations, or those of the publisher, the editors and the reviewers. Any product that may be evaluated in this article, or claim that may be made by its manufacturer, is not guaranteed or endorsed by the publisher.

## Footnotes

^1^ http://pymol.sourceforge.net

^2^ https://www.uniprot.org/

^3^ https://www.rcsb.org
