# Supplementary material for: Structural insights into catalytical capability for CPT11 hydrolysis and substrate specificity of a novel marine microbial carboxylesterase, E93
Source: Front Microbiol. 2023 Jan 11;13:1081094. doi: 10.3389/fmicb.2022.1081094 (PMC9901791; doi:10.3389/fmicb.2022.1081094)
Supplement: Supplementary file 1 [file Data_Sheet_1.PDF]

Supplementary materials

Figure S1. Polygenetic analysis of E93

Phylogenetic tree based on amino acid sequences of E93 and its homologous proteins. Sequence alignment was performed using ClustalW. The phylogenetic tree was constructed by MEGA software. Bootstrap values were based on 1,000 replicates and the values higher than 50 % were shown in the tree. The scale bar measured the number of amino acid substitutions per site. E93 highlighted by red box.

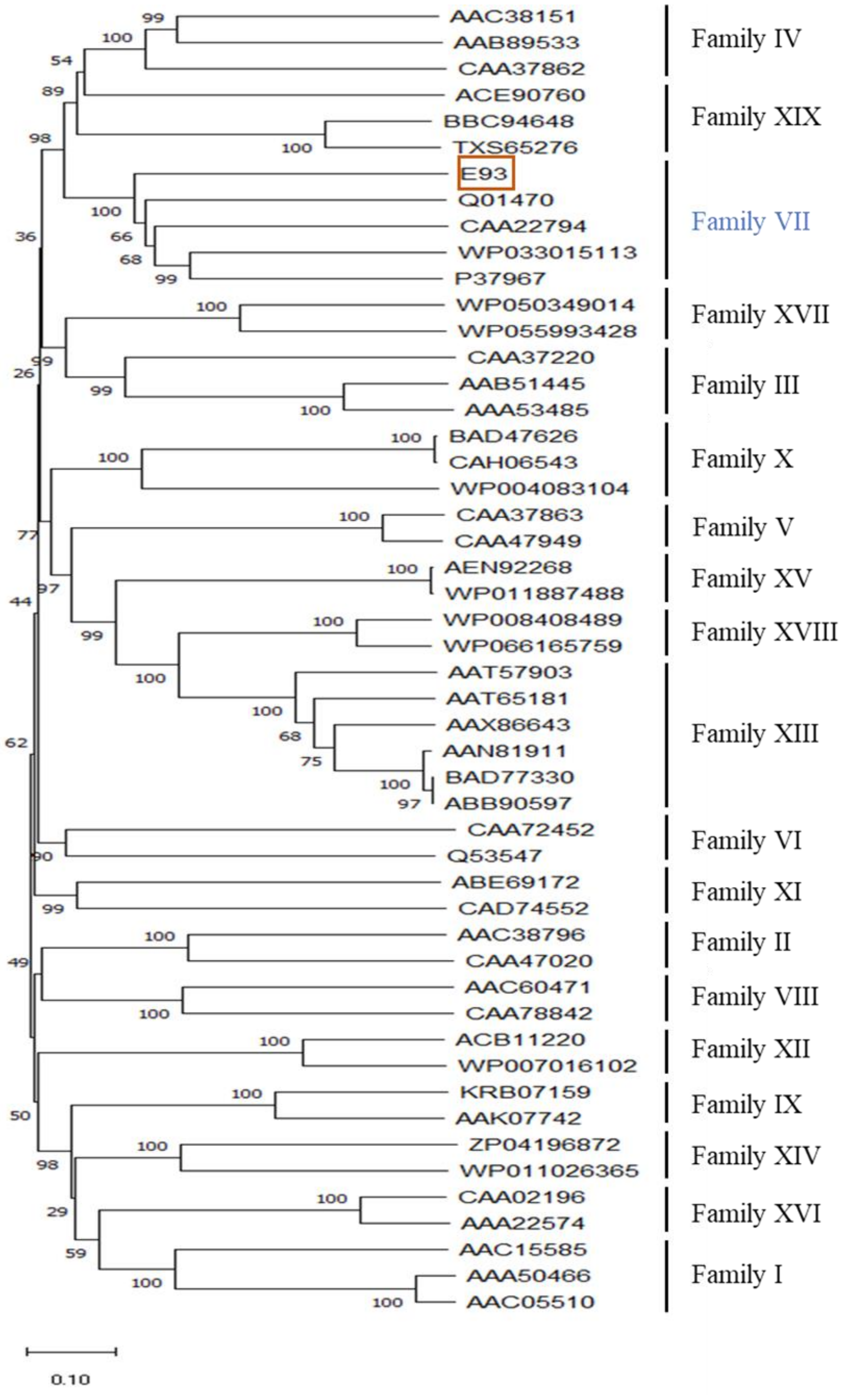

Figure S2. Multiple sequence alignment of E93 with similar proteins

Amino acids conserved in all sequences used for alignment were labeled with the red-colored background. Comparative conserved amino acids were labeled with red color. The catalytic triad is highlighted by the red pentagrams. The mutated sites were highlighted with blue frame, and the catalytic triad were highlighted with black frame.

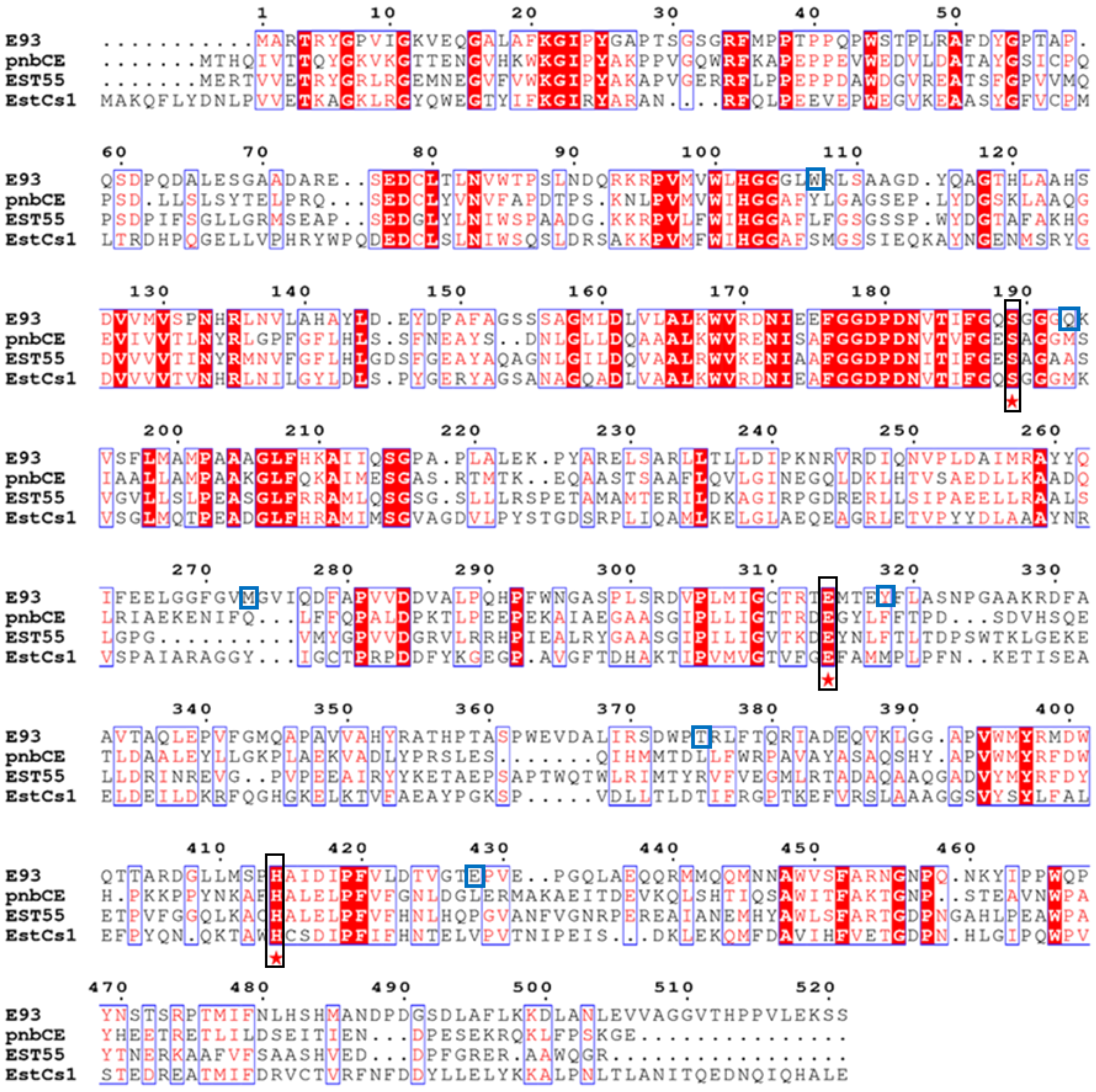

Table S1: Data collection of E93

Item	E93
PDB No.	7X8L
**Data collection**	
Wavelength	0.97923
Resolutions (Å)	35.32 - 1.77 (1.833 - 1.77) [a]
Space group	P 2_1 2_1 2_1
Unit cell (Å, °)	a = 44.85 b = 91.67 c = 110.82
	α=β=γ=90
Unique reflections	45256 (4433)
Completeness (%)	99.65 (99.19)
Mean I/σ (I)	23.91 (6.44)
R-merge [b]	0.09239 (0.6847)
R-meas	0.09595 (0.7094)
R-pim	0.02559 (0.1844)
CC1/2	0.999 (0.966)
**Refinement statistics**	
Reflections used in refinement	45246 (4431)
R-work [c]	0.1678 (0.2032)
R-free [d]	0.1924 (0.2470)
No. atoms (protein)	3957
No. atoms (water)	274
**RMSD**	
Bond lengths (Å)	0.008
Bond angles (°)	1.02
Ramachandran favored (%)	96.45
Ramachandran allowed (%)	3.55
Ramachandran outliers (%)	0
Average B-factor (Å2)	20.97

[a] Statistics for the highest-resolution shell are shown in parentheses.

[b] R_{merge}=Σ |Ii − < I> |/Σ |I|, where I_i is the intensity of an individual reflection and is the average intensity of that reflection.

[c] R_{work}=Σ ||Fo| − |Fc||/Σ |Fo|, where F_o and F_c are the observed and calculated structure factors for reflections, respectively.

[d] R_{free} was calculated as R_{work} using the 5 % of reflections that were selected randomly and omitted from refinement.

Table S2: Hydrolysis of prodrug CPT11 by E93 and mutants

Enzyme	Substrate	***K*m**	***K*cat**	***K*cat/*K*m**
		mM	10^{-3} min^{-1}	min^{-1} mM^{-1}
E93		70.17 ± 7.07	40.08 ± 1.22	0.57 ± 0.06
E93_W107A		59.63 ± 4.37	104.51 ± 8.19	1.75 ± 0.22
E93_M273A		58.05 ± 5.3	77.29 ± 3.20	1.33 ± 0.15
E93_Q193A		81.01 ± 8.44	5.41 ± 0.31	0.067 ± 0.01
E93_Y318A	CPT11	73.61 ± 7.25	20.26 ± 1.03	0.28 ± 0.04
E93_Y318F		73.46 ± 7.22	22.02 ± 1.12	0.30 ± 0.04
E93_T375A		74.20 ± 8.70	19.10 ± 1.75	0.26 ± 0.05
E93_T375I		75.39 ± 7.33	21.45 ± 2.04	0.28 ± 0.05
E93_E428A		69.62 ± 5.79	40.01 ± 2.77	0.57 ± 0.1
E93_S189A		N.D.[a]	N.D.[a]	N.D.[a]

[a] No detectable hydrolysis.

Table S3: Hydrolysis of NPC by E93 and mutants

Enzyme	Substrate	***K*m**	***K*cat**	***K*cat/*K*m**
		μM	10^{-3} min^{-1}	min^{-1} mM^{-1}
E93		75.66 ± 6.03	35.40 ± 1.70	0.47 ± 0.05
E93_W107A		63.11 ± 5.73	44.81± 2.17	0.71 ± 0.08
E93_M273A		66.11 ± 4.12	70.94 ± 6.79	1.07 ± 0.10
E93_Q193A		71.29 ± 5.35	30.69 ± 2.27	0.43 ± 0.05
E93_Y318A	NPC	55.47 ± 5.17	40.87± 3.07	0.74± 0.10
E93_Y318F		76.62 ± 8.19	31.52 ± 2.05	0.41 ± 0.06
E93_T375A		75.09 ± 7.92	32.16 ± 2.86	0.43 ± 0.07
E93_T3751		75.14 ± 7.16	31.07 ± 2.03	0.41 ± 0.03
E93_E428A		85.07 ± 9.4	23.80 ± 2.34	0.28 ± 0.05
E93_S189A		N.D.[a]	N.D.[a]	N.D.[a]

[a] No detectable hydrolysis.

Table S4: Hydrolysis of *p*-NP hexanoate (C6) by E93 and mutants

Enzyme	Substrate	*Km* (*mM*)	*Kcat* ($\times 10^5\ S^{-1}$)	*Kcat*/*Km* ($\times 10^6\ S^{-1}mM^{-1}$)
E93	C6	0.18 ± 0.011	4.31 ± 0.32	2.34 ± 0.23
E93_W107A		0.21 ± 0.018	3.48 ± 0.15	1.63 ± 0.09
E93_M273A		0.39 ± 0.025	3.06 ± 0.08	0.78 ± 0.20
E93_Q193A		0.10 ± 0.01	12.86 ± 1.01	12.49 ± 0.13
E93_Y318A		0.18 ± 0.02	3.96 ± 0.19	2.20 ± 0.15
E93_Y318F		0.19 ± 0.02	4.00 ± 0.27	2.16 ± 0.11
E93_T375A		0.13 ± 0.01	7.99 ± 0.41	6.01 ± 0.21
E93_T375I		0.11 ± 0.01	8.22 ± 0.43	7.51 ± 0.12
E93_E428A		0.18 ± 0.02	4.29 ± 0.21	2.42 ± 0.08
E93_S189A		N.D.[a]	N.D.[a]	N.D.[a]

[a] No detectable hydrolysis.

Table S5: Hydrolysis of *p*-NP butyrate (C4) by E93 and mutants of Thr375

Enzyme	Substrate	*Km* (*mM*)	*Kcat* ($\times 10^5\ S^{-1}$)	*Kcat*/*Km* ($\times 10^6\ S^{-1}mM^{-1}$)
E93	C4	0.21 ± 0.02	3.38 ± 0.11	1.65 ± 0.33
E93_T375I		0.10 ± 0.01	9.76 ± 0.63	9.48 ± 0.80

Table S6: Primers for E93 and mutants

Primer	Sequence
E93-F	tcgcggatccatggcccgcactcgctatg
E93-R	atttgcggccgctcatgaagacttctccaatacg
E93_W107A-F	cggcggcctggctcgcttg
E93_W107A-R	ccgacaagcgagccaggccg
E93_M273A-F	gcttcggtgtcgctggagtgat
E93_M273A-R	aatcctgaatcactccagcgacacc
E93_Q193A-F	ggcgggggagcgaaagtgtc
E93_Q193A-R	ggaaggacactttcgctccccc
E93_Y318A-F	aatgaccgaggctttcctggccagc
E93_Y318A-R	gccaggaaagcctcggtcatttcgg
E93_Y318F-F	aatgaccgagtttttcctggccagc
E93_Y318F-R	gctggccaggaaaaactcggtcatt
E93_T375A-F	tccgactggccagcccggctgttca
E93_T375A-R	tgaacagccgggctggccagtcgga
E93_T375I-F	tccgactggccaatccggctgttca
E93_T375I-R	tgaacagccggattggccagtcgga
E93_E428A-F	acggtcggcaccgcaccggtcgagc
E93_E428A-R	ctcgaccggtgcggtgccgaccgtgt
